# Development and validation of a machine learning model based on multiple kernel for predicting the recurrence risk of Budd-Chiari syndrome

**DOI:** 10.3389/fphys.2025.1589469

**Published:** 2025-05-30

**Authors:** Weirong Xue, Bing Xu, Hui Wang, Xiaoxiao Zhu, Jiajia Qin, Guangshuang Zhou, Peilin Yu, Shengli Li, Yingliang Jin

**Affiliations:** ^1^ School of Public Health, Xuzhou Medical College, Xuzhou, China; ^2^ Department of Otorhinolaryngology, Affiliated Hospital of Xuzhou Medical University, Xuzhou, Jiangsu, China; ^3^ Department of Hepatobiliary Surgery, Xuzhou Central Hospital, Xuzhou, Jiangsu, China; ^4^ Clinical Research Institute, The Affiliated Hospital of Xuzhou Medical University, Xuzhou, China; ^5^ Department of Biostatistics, Center for Medical Statistics and Data Analysis, School of Public Health, Key Laboratory of Human Genetics and Environmental Medicine Xuzhou Medical College, Xuzhou, China

**Keywords:** Budd-Chiari syndrome, recurrence, machine learning, multiple kernel learning, predict

## Abstract

**Background:**

Budd-Chiari syndrome (BCS) is a rare global condition with high recurrence rates. Existing prognostic scoring models demonstrate limited predictive efficacy for BCS recurrence. This study aims to develop a novel machine learning model based on multiple kernel learning to improve the prediction of 3-year recurrence in BCS patients.

**Methods:**

Data were collected from BCS patients admitted to the Affiliated Hospital of Xuzhou Medical University between January 2015 and July 2022. The dataset was divided into training, validation, and test sets in a 6:2:2 ratio. Models were constructed by evaluating all combinations of four kernel functions in the training set. Hyperparameters for each model were optimized using the particle swarm optimization (PSO) algorithm on the validation set. The test set was used to compare kernel function combinations, with the area under the curve (AUC), sensitivity, specificity, and accuracy as evaluation metrics. The optimal model, identified through the best-performing kernel combination, was further compared with three classical machine learning models.

**Result:**

A kernel combination integrating all four basic kernels achieved the highest average AUC (0.831), specificity (0.772), and accuracy (0.780), along with marginally lower but more stable sensitivity (0.795) compared to other combinations. When benchmarked against classical machine learning models, our proposed MKSVRB (Multi-Kernel Support Vector Machine Model for Three-Year Recurrence Prediction of Budd-Chiari Syndrome) demonstrated superior performance. Additionally, it outperformed prior studies addressing similar objectives.

**Conclusion:**

This study identifies risk factors influencing BCS recurrence and validates the MKSVRB model as a significant advancement over existing prediction methods. The model exhibits strong potential for early detection, risk stratification, and recurrence prevention in BCS patients.

## Introduction

Budd-Chiari syndrome (BCS) is defined as the obstruction of hepatic venous outflow at any level ranging from the small hepatic venules to the junction of the inferior vena cava and right atrium, caused by factors other than hepatic venous occlusive disease and cardiac disease (Janssen et al., 2003). Common causes typically include hypercoagulable states, infections, and malignant tumors (Goel et al., 2015). This obstruction leads to increased hepatic sinusoidal pressure and portal venous pressure, reduced blood flow, resulting in hepatic congestion and ascites formation. Prolonged hepatocyte hypoxia can result in hepatocellular injury, potentially leading to cirrhosis and portal hypertension (Menon et al., 2004; Valla, 2002).Budd–Chiari syndrome, is globally rare and exhibits significant geographical variations in etiology, incidence, and clinical presentation. Chronic cases are more prevalent, while acute occurrences are uncommon. Typical features include abdominal pain, ascites, hepatomegaly, and subcutaneous vascular dilatation in the abdominal wall and trunk (Shukla et al., 2021). Advances in endovascular therapies have significantly improved patient prognosis, with a sustained decrease in mortality rates. However, The phenomenon of recurrence still occurs frequently (Shukla et al., 2021). Various prognostic scoring models exist including Child–Pugh score, Model for end-stage liver disease (MELD), Clichy PI, Rotterdam score, New Clichy PI, and BCS-TIPS score, while these models have shown limited predictive efficacy for recurrence of Budd–Chiari syndrome (Wang et al., 2023).

In a study by Zhongkai Wang et al., a model for predicting BCS recurrence based on logistic regression (LR) and nomograms demonstrated superior performance compared to traditional scoring models (Wang et al., 2023). With the rapid development of machine learning technology, its application in various industries has expanded significantly. In the field of medicine, using machine learning techniques to predict the diagnosis, mortality, and prognosis of various diseases has become feasible. The potential machine learning in predicting Budd-Chiari syndrome recurrence as a more accurate and efficient predictive tool is promising (Deo, 2015).

In the field of machine learning, common algorithms used for predicting disease mortality or recurrence include random forest (RF), support vector machine (SVM), extreme gradient boosting (XGBoost), and LR models. These algorithms generally perform well when the dataset exhibits relatively simple structural relationships or minimal internal noise (Handelman et al., 2018; Jamin et al., 2021). However, when the internal complexity of the dataset is high, there are many noise and outliers, or the relationships between datasets are difficult to represent with a single logical relationship, these machine learning algorithms typically fail to achieve satisfactory performance.For SVM, its performance largely depends on the choice of kernel (Amari and Wu, 1999), while multiple kernel learning (MKL) is a machine learning method that combines multiple kernel functions or selects the optimal kernel function to enhance the performance and generalization ability of the model. Compared to traditional machine learning models, MKL can utilize multiple kernel functions to measure the similarity between samples, aiming to describe the internal relationships within the dataset as comprehensively as possible. This approach better captures the complexity inherent in the dataset, thereby improving the utilization of data (Sonnenburg et al., 2006),When confronted with classification and regression problems involving heterogeneous datasets from various sources, multiple kernel learning has been proven to be an effective solution. It finds wide applications in many fields, including visual object recognition (Bucak et al., 2014), early disease identification (Collazos-Huertas et al., 2019), disease prognosis prediction (Wilson et al., 2019) and more.

Budd-Chiari syndrome is characterized by its rarity, making it challenging to obtain ample sample sizes in most studies. Moreover, there are significant regional differences and complex etiologies involved, most obstructions occur in the hepatic veins and the segment of the inferior vena cava above their openings can lead to the syndrome (Menon et al., 2004). The characteristics of Budd-Chiari syndrome outlined above necessitate a broader consideration when establishing predictive models, especially for features not universally common due to rare causative factors. Baseline data explained solely by a single-kernel model often fails to provide reliable guidance on recurrence. MKL, as a method capable of effectively handling heterogeneous data sources and noisy datasets, should yield satisfactory results when applied to predicting recurrence in Budd-Chiari syndrome (Pavlidis et al., 2001; Kingsbury et al., 2005).

However, up to this point, MKL as a powerful descriptive tool has not been applied to predict recurrence in Budd-Chiari syndrome. Therefore, in this paper, we will establish a new supported vector machine model with MKL for feature learning and particle swarm optimization (PSO) algorithm for hyperparameter selection, in the purpose of predicting recurrence in Budd-Chiari syndrome within 3 years.

## Methods

### Data source and study population

The dataset was obtained from patients diagnosed with BCS admitted to the Affiliated Hospital of Xuzhou Medical University between January 2015 and July 2022. Inclusion criteria were based on symptoms, signs, and imaging examinations indicating primary BCS, including magnetic resonance imaging (MRI), computed tomography (CT), color Doppler ultrasound (CDUS), and venography. Exclusion criteria were as follows: (1) patients with secondary BCS caused by various reasons, including parasitic invasion, abscess, cyst, malignant tumor compression, or venous injury after surgery; (2) patients with concurrent liver diseases, including viral hepatitis, autoimmune hepatitis, alcoholic hepatitis, and liver fibrosis; (3) patients with severe cardiac, hepatic, or renal failure, or other reasons unable to undergo interventional or surgical treatment; (4) patients who failed in revascularization due to complete vascular occlusion or concomitant old thrombosis; (5) patients with improper anticoagulation therapy; (6) patients with significant missing information in medical records; (7) patients with follow-up time less than 12 months. According to these criteria, a total of 522 patients were included in this study, with complete data availability and no missing values.

This study was approved by the Institutional Review Board of the Affiliated Hospital of Xuzhou Medical University. All methods were performed in accordance with the relevant guidelines and regulations.

### Model framework

Before construsting the model, the feature data of Budd-Chiari syndrome patients is required to be represented in the following form: $T=\left\{{\left(x_{i\;},y_{i}\right)\;}_{i=1}^{N}\right\}$ ,Among them, $x_{i}\in R^{d}$ is the patient’s feature vector, $y_{i}\in \left\{-1,+1\right\}$ is the label indicating whether the patient with Budd-Chiari syndrome has relapsed, $y_{i}=+1$ represents the patient relapsing within 3 years, while $y_{i}=-1$ represents the patient not relapsing within 3 years. $k_{m}\left(x_{i\;},x_{j}\right)=\langle \phi \left(x_{i}\right),\phi \left(x_{j}\right)\rangle$ with $x_{i\;},x_{j}\in R^{d}$ is defined as the method to handle data that is linearly inseparable in low-dimensional space, known as the kernel trick, where $\phi \left(x\right):R^{d}\to R^{D}$ is the mapping function that maps the data, which is linearly inseparable in low-dimensional space ${\;R}^{d}$ , to high-dimensional space ${\;R}^{D}$ , and $k_{m}\left(x_{i\;},x_{j}\right)$ is referred to as the m -th kernel function. Explicitly calculating the inner product of data mapped to high-dimensional space is typically computationally challenging. The kernel trick allows for the implicit computation of the inner product in high-dimensional space by computing the kernel function, significantly reducing storage space and computational costs (Lanckriet et al., 2004; Scholkopf et al., 1999).

A composite kernel function is then established based on the weights of different kernel functions:

$$k\left(x_{i\;},x_{j}\right)=\sum_{m=1}^{L}\eta_{m}k_{m}\left(x_{i\;},x_{j}\right)=\sum_{m=1}^{L}\eta_{m}\phi_{m}{\left(x_{i}\right)}^{T}\phi_{m}\left(x_{j}\right)$$ (1)

In Equation 1 L is the number of kernels involved in model and $\eta_{m}$ represents the weight coefficients of each kernel function in the composite kernel function, while the sum of the weight coefficients of all kernel functions $\sum_{m=1}^{L}\eta_{m}=1$ . In this paper, we discuss four types of kernel functions, therefore L=4 .

Then the EasyMKL algorithm, proposed by Fabio Aiolli and Michele Donini in 2015, was used to obtain the weights of each base kernel in the kernel set by solving a simple QP problem with the learning strategy that considering the balance between the minimum and average values of the boundary (Aiolli and Donini, 2015; Donini et al., 2019; Aiolli et al., 2008):

$$\max_{\eta }\;\min_{\gamma }\;\left(1-\varphi \right)\gamma^{T}Y\left(\sum_{m=1}^{L}\eta_{m}K_{m}\right)Y\gamma +\varphi {\left\|\gamma \right\|}_{2}^{2}$$ (2)

In the above equation, a trade-off parameter φ is defined as $\varphi \in \left[0,1\right]$ , $Y=\left\{y_{i}\left|1\leq i\leq N\right.\right\}$ and K are kernel matrices formed by the kernel function values between training samples, obviously $K\in R^{N\times N}$ . The probability vector $\gamma \in R^{N}$ represents a collection of probabilities for each sample being selected among all positive or negative instances. Due to $\sum_{i\in ⊕}\gamma_{i}=1$ and $\sum_{i\in ⊖}\gamma_{i}=1$ , while i∈⊕ represents all positive samples, i∈⊖ represents all negative samples, ${\left\|\gamma \right\|}_{1}=2$ can be clearly obtained. η is called the weight vector, defined as the set of weight coefficients of all the kernel function, $\eta =\left\{\eta_{m}|m=1,2,......,L\right\}$ .

Then, for Equation 2, a iteration process is employed to optimize the objective function. In each optimization step, the algorithm selects a pair of variables $\left(\gamma_{r\;},\gamma_{q}\right)$ , keeping other variables fixed, and iteratively updates these two probability variables to compute their optimal solution. This process continues until convergence is achieved and resulting in the global optimal solution.

$$\gamma_{r}^{\prime }\leftarrow \gamma_{r}+\epsilon ,{\;\gamma }_{q}^{\prime }\leftarrow \gamma_{q\;}-\epsilon$$ (3)

In Equation 3, ϵ is the change in a pair of variables $\left(\gamma_{r\;},\gamma_{q}\right)$ selected during each update, it is necessary for the optimal solution that the partial derivative of $\nabla_{L\left(\gamma \right)}=L\left(\gamma^{\prime }\right)-L\left(\gamma \right)$ when the objective function in Equation 2 defined as $L\left(\gamma \right)$ with respect to ϵ is equal to zero:

$$\frac{\partial \nabla_{L\left(\gamma \right)}}{\partial \epsilon }=0$$ (4)

In Equation 4, the solution for ϵ within the feasible domain is obtained, followed by acquiring the probability vector γ and weight vector η .

In our study, four common kernel functions were selected as alternative options for support vector machines. The kernel functions are as follows:

$$\text{Linear Kernel}:\;k_{1}\left(x_{i\;},x_{j}\right)=\langle x_{i},x_{j}\rangle =x_{i}^{T}x_{j}$$ (5a)

$$\text{Polynomial Kernel}:\;k_{2}\left(x_{i\;},x_{j}\right)={\left(x_{i}^{T}x_{j}+offset\right)}^{degree}$$ (5b)

$$\text{Sigmoid Kernel}:\;k_{3}\left(x_{i\;},x_{j}\right)=\tanh\left(\alpha x_{i}^{T}x_{j}+\beta \right)$$ (5c)

$$\text{Gaussian Kernel}:\;k_{4}\left(x_{i\;},x_{j}\right)=\exp\left(-\sigma {\left\|x_{i}-x_{j}\right\|}^{2}\right)$$ (5d)

In these functions, offset is a constant term, degree is the degree of the polynomial, α is a scale parameter affecting the extent of data transformation in the non-linear space, constant β adjusts the bias of the Sigmoid kernel function, and parameter σ controls the width of the Gaussian kernel function.

Finally considering how to optimize the aforementioned parameters and trade-off parameter φ , PSO algorithm is an optimization algorithm proposed by James Kennedy and Russell Eberhart in 1995 (Kennedy and Eberhart, 1995).By simulating the process of foraging in bird flocks and fish schools in nature, leveraging the collaboration of a group to find optimal solutions to problems, PSO algorithm offers advantages such as requiring fewer parameters to be tuned and being easy to implement. Today, it has been widely employed in various fields including geophysics (Pace et al., 2021), network data classification (Carneiro et al., 2019), energy engineering (Ali Ahmadi et al., 2013) and others for optimization problems.

After predefining a set of kernel combinations as inputs, the position of the h -th particle in the l -th iteration within the Z -dimensional search space is denoted as $W_{h}^{l}=\left(w_{h,1}^{l},w_{h,2}^{l},w_{h,3}^{l},...,w_{h,Z}^{l}\right)$ , where both the number of particles and maximum iterations are defined as 10 during the optimization process. The parameter Z corresponds to the total number of hyperparameters requiring optimization, including the Polynomial kernel’s offset and degree , the Sigmoid kernel’s α and β , the Gaussian kernel’s σ , and a universal trade-off parameter φ shared across all kernel combinations.

In the particle swarm optimization process, the distance and direction of movement for the h -th particle during the l -th iteration are represented as $V_{h}^{l}=\left(v_{h,1}^{l},v_{h,2}^{l},v_{h,3}^{l},...,v_{h,Z}^{l}\right)$ . The historical best position of the h -th particle is denoted as ${pb}_{h}=\left(p_{h,1},p_{h,2},p_{h,3},...,p_{h,Z}\right)$ , while the global historical best position across all particles is denoted as $gb=\left(p_{1},p_{2},p_{3},...,p_{Z}\right)$ . During each iteration, the spatial position of the h -th particle is updated based on its position from the previous iteration, combined with the current iteration’s movement direction and distance.

$$W_{h}^{l+1}=W_{h}^{l}+v_{h}^{l+1}$$ (6)

The direction and distance of movement in Equation 6 in each iteration are determined by three factors: the direction and distance from the previous iteration, the particle’s historical best position, and the swarm’s global historical best position:

$$v_{h}^{l+1}=\psi v_{h}^{l}+c_{1}r_{1}\left({pb}_{h}-{\;W}_{h}^{l}\right)+c_{2}r_{2}\left(gb-W_{h}^{l}\right)$$ (7)

The first part in Equation 7 is the inertial component, which encourages particles to retain their previous motion state, governed by the inertia weight ψ . This parameter reflects the particle’s confidence in its prior trajectory. The second component is the cognitive term, directing particles toward their individual historical best positions, quantified by the individual learning factor $c_{1}$ , which determines the reliance on the particle’s own exploration history. The third component is the social term, guiding particles toward the swarm’s global historical best position, regulated by the social learning factor $c_{2}$ , representing the influence of collective swarm knowledge. In the modeling process, ψ , $c_{1}$ , and $c_{2}$ are empirically specified as 0.9, 2, and 2, respectively. Random numbers $r_{1}$ and $r_{2}$ ,which uniformly sampled between 0 and 1, were incorporated to balance exploration-exploitation trade-offs between individual and group experiences.

The optimization objective focused on maximizing the validation set AUC. For each particle in every iteration, hyperparameters were systematically recorded, and the configuration yielding the highest AUC was selected as the optimal hyperparameter set for the corresponding kernel combination.

### Study design

Budd-Chiari syndrome patients included in the study were randomly divided into training, validation, and testing sets in a 6:2:2 ratio. The training set was utilized for the model to learn the data features. After determining a suitable set of hyperparameters using the PSO algorithm, a model associated with these hyperparameters was constructed, and the validation set was used to evaluate the model’s performance to select the best hyperparameter combination.

**FIGURE 1:**
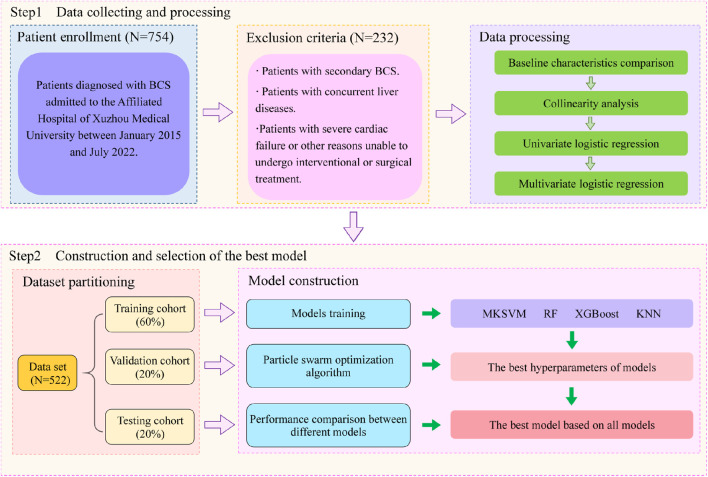
Flow chart of study design.

In many previous studies on multi-kernel learning, specifying kernel combinations was typically empirical. Jian Hou et al. proposed that a linear combination of more kernels may not necessarily be superior to the average combination of single strong kernels or base kernels (Hou et al., 2018). To clearly observe the measuring ability of different kernel functions for sample similarity and determine the best kernel combination, we arranged combinations of four base kernels. The testing set was used to compare the performance of a total of four single-kernel support vector machine models and eleven multi-kernel models. Additionally, three classical machine learning models, including RF, XGBoost, and KNM, were also considered. Ultimately, a total of eighteen models were included in this study for the evaluation of predictive performance for BCS patient recurrence. Ten rounds of validation were conducted on each of the aforementioned models, and the evaluation was performed using the average performance metrics and standard deviation, including AUC (area under the curve), sensitivity, specificity and accuracy.The flowchart depicted in Figure 1.

### Statistical analysis

In the statistical analysis stage, we conducted baseline analysis of the dataset to detect baseline feature differences between BCS patients who experienced recurrence during the observation period and those who did not. For categorical variables, we described the number and composition of each category in the recurrence and non-recurrence groups, and employed the chi-square test to identify differences between the two groups. For continuous variables, we first conducted tests for normality in both groups. For variables following a normal distribution, we described them using mean and standard deviation and then used independent samples t-test to identify differences. For variables not following a normal distribution, we described them in terms of median and IQR and then conducted the non-parametric Mann-Whitney U test.

After determining the variables with statistically significant differences in baseline analysis, a univariate logistic regression analysis was conducted to examine the relationship between various data features and the recurrence of BCS, and to identify factors that may affect patient recurrence. Subsequently, a multivariate logistic analysis was performed to determine the independent risk factors affecting recurrence and their corresponding risk levels. In this study, all statistical analyses were considered statistically significant at P < 0.05. The software used for analysis included R (version 4.4.1) and Python (Version 3.10.9).

## Results

### Baseline characteristics

**FIGURE 2:**
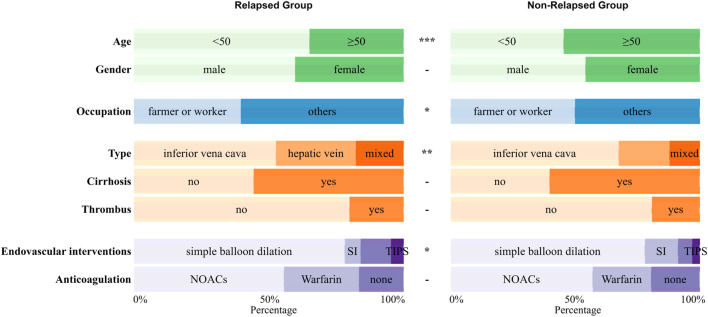
The distribution of qualitative variables between the relapsed group and the non-relapsed group with significance marked between the two groups (p ≥ 0.05 -; p < 0.05 *; p < 0.01 **; p < 0.001 ***).

**FIGURE 3:**
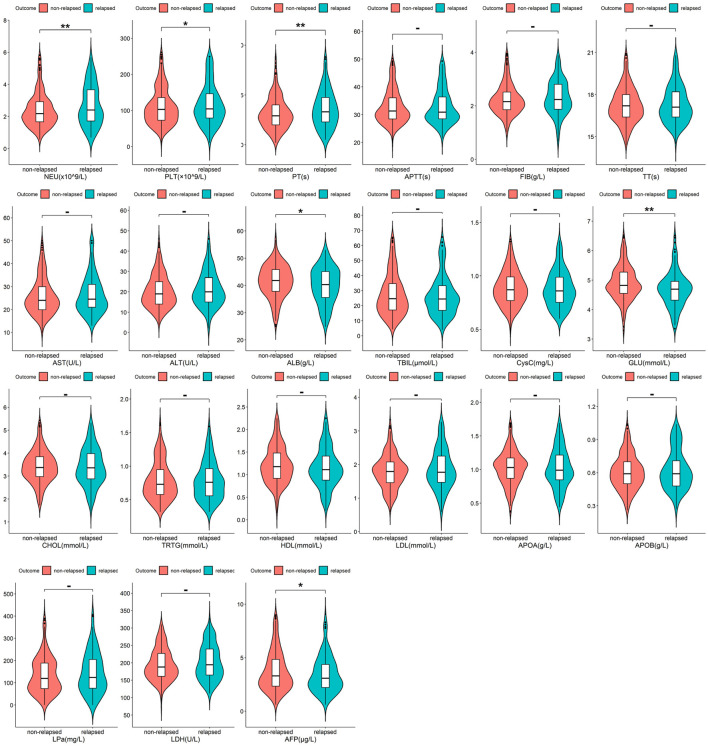
The distribution of quantitative variables between the relapsed group and the non-relapsed group with significance marked between the two groups (p ≥ 0.05 -; p < 0.05 *; p < 0.01 **; p < 0.001 ***).

Among all 522 Budd-Chiari syndrome patients included in the study, 169 experienced recurrence during the observation period, while 353 did not (1:2.09). Valid data obtained in this study include patients’ age, gender, occupation, Budd-Chiari syndrome subtype, and other information, as detailed in Figure 2 and Figure 3.

**FIGURE 4:**
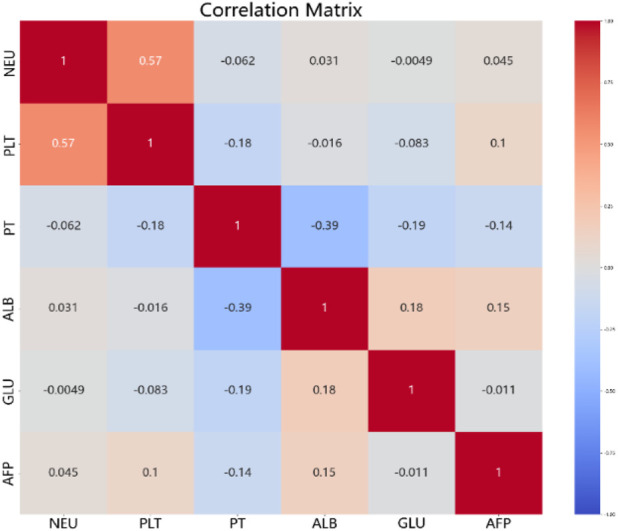
The Correlation Matrix of quantitative variables after baseline analysis.

As shown in Figure 2 and Figure 3, Budd-Chiari syndrome patients who experienced recurrence during the observation period differed significantly from those who did not in terms of age (P < 0.001), occupation (P = 0.029), type (P = 0.005), endovascular intervention (P = 0.01), Neutrophils (NEU) levels (P = 0.002), Platelets (PLT) levels (P = 0.044), Prothrombin Time (PT) levels (P = 0.007), Albumin (ALB) levels (P = 0.02), Glucose (GLU) levels (P = 0.004), and Alpha-Fetoprotein (AFP) levels (P = 0.032). We conducted collinearity analysis among these variables in Figure 4, revealing strong collinearity between NEU and PLT, while no significant collinearity was found among the other variables. Considering the significance of the differences, we decided to exclude PLT.

### Feature selection

The results of univariate and multivariate screening are presented in Table 1. In the univariate screening, eight variables were identified, including age (P < 0.001), occupation (P = 0.029), type (compared to inferior vena cava type, hepatic vein type with P = 0.005, mixed type with P = 0.02), endovascular intervention (compared to simple balloon dilation, stent implantation with P = 0.025, catheter-directed thrombolysis with P = 0.043), NEU (P = 0.001), PT (P = 0.031), ALB (P = 0.01), and AFP (P = 0.022).

**TABLE 1:** Univariate and multivariate regression analysis of risk factors for recurrence of Budd-Chiari syndrome.

Characteristic	Univariate analysis			Multivariate analysis		
	OR	95%CI	P	OR	95%CI	P
Age						
≤50	ref	ref	ref	ref	ref	ref
>50	0.445	0.304–0.650	**<0.001**	0.419	0.280–0.627	**<0.001**
Occupation						
Others	ref	ref	ref			
Farmer or worker	0.661	0.455–0.958	**0.029**			
Type						
Inferior vena cava	ref	ref	ref			
Hepatic vein	1.857	1.202–2.870	**0.005**			
Mixed	1.866	1.103–3.157	**0.02**			
Endovascular intervention						
Simple balloon dilation	ref	ref	ref	ref	ref	ref
Stent implantation	0.443	0.217–0.905	**0.025**	0.419	0.200–0.878	**0.021**
Catheter-directed thrombolysis	1.979	1.022–3.834	**0.043**	2.57	1.273–5.188	**0.008**
TIPS	1.515	0.595–3.856	0.383	0.809	0.287–2.279	0.689
NEU	1.21	1.078–1.358	**0.001**	1.197	1.062–1.349	**0.003**
PT	1.046	1.004–1.090	**0.031**			
ALB	0.963	0.936–0.991	**0.01**	0.967	0.937–0.997	**0.033**
GLU	0.992	0.874–1.125	0.899			
AFP	0.919	0.855–0.988	**0.022**	0.92	0.854–0.990	**0.025**

The bolded text represents variables with P value <= 0.05.

The results of multivariable logistic regression screening indicated that age ≥50 years may be a protective factor for Budd-Chiari syndrome patients compared to age <50 years (P < 0.001, OR = 0.419), which is consistent with the findings of Wang et al. (2023). Higher NEU levels were identified as an independent risk factor for Budd-Chiari syndrome recurrence (P = 0.003, OR = 1.197), while higher levels of ALB (P = 0.033, OR = 0.967) and AFP (P = 0.025, OR = 0.92) were found to have protective effects against Budd-Chiari syndrome recurrence.Among the various endovascular interventions for Budd-Chiari syndrome, patients undergoing stent implantation seemed to have a lower risk of recurrence compared to those undergoing simple balloon dilation (P = 0.021, OR = 0.419). However, patients undergoing catheter-directed thrombolysis showed a higher tendency for recurrence (P = 0.008, OR = 2.57).

### Selection of the best combination of kernel functions

**FIGURE 5:**
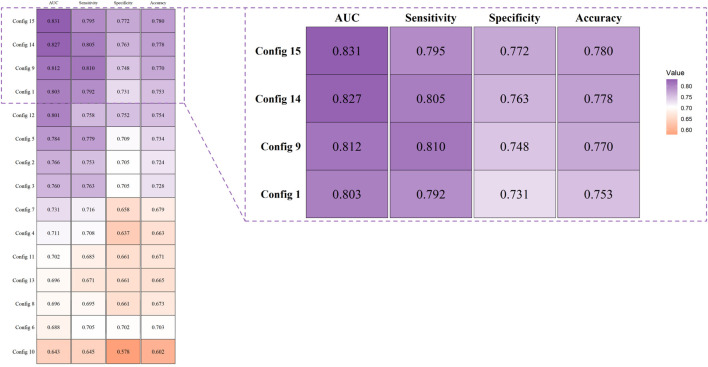
Evaluation and comparison of models were conducted using AUC, accuracy, sensitivity, and specificity.

In our study, we constructed support vector machine models based on fifteen combinations of four types of kernel functions, including four single-kernel models and eleven multi-kernel models. The combinations of kernel functions are listed in Table 2. Each combination model underwent ten rounds of validation and the evaluation was based on the average and standard deviation of ten results, including metrics such as AUC, sensitivity, specificity, and accuracy, as detailed in Figure 5 and Table 2. It can be observed that among all four base kernels, the Linear Kernel demonstrated the best fit for our Budd-Chiari syndrome patient data and achieved the highest average AUC, sensitivity, specificity, and accuracy while Gaussian Kernel having the poorest average performance.Surprisingly, in terms of kernel combinations, Config 9, which combines the Polynomial Kernel with the Gaussian Kernel—the two weaker kernels among the four base kernels—outperformed Config 6, which combines the Linear Kernel and the Sigmoid Kernel, both of which had higher individual performances among the single kernels. This finding demonstrates the effectiveness of our study, suggesting that combinations of multiple weaker kernels may outperform single strong kernels. For configurations with more kernels, the three-kernel classifier combinations of Config 12 and Config 14 exhibited stronger performance and stability. However, the four-kernel classifier was deemed optimal, as it demonstrated the most comprehensive data descriptive and learning capabilities. Despite Config 9 surpassing Config 15 in average sensitivity, considering overall stability, the four-kernel classifier was deemed to have the most robust performance.

**TABLE 2:** The Fifteen combinations of kernel functions and mean and standard deviation of indexs of fifteen kernel function combinations in ten rounds of validation.

Combinations	Kernel functions	AUC	Sensitivity (%)	Specitivity (%)	Accuracy (%)
Config 1	$k_{1}$	0.8030 ± 0.0479	79.21 ± 7.89	73.14 ± 4.93	75.34 ± 5.47
Config 2	$k_{2}$	0.7660 ± 0.0731	75.27 ± 9.46	70.48 ± 6.53	72.38 ± 6.58
Config 3	$k_{3}$	0.7600 ± 0.0867	76.32 ± 9.28	70.48 ± 6.53	72.76 ± 7.35
Config 4	$k_{4}$	0.7110 ± 0.0399	70.79 ± 6.01	63.73 ± 5.17	66.29 ± 4.88
Config 5	$k_{1}+k_{2}$	0.7840 ± 0.0753	77.89 ± 9.22	70.90 ± 6.10	73.43 ± 7.05
Config 6	$k_{1}+k_{3}$	0.6880 ± 0.1054	70.53 ± 12.82	70.15 ± 7.52	70.29 ± 7.90
Config 7	$k_{1}+k_{4}$	0.7310 ± 0.0493	71.58 ± 6.30	65.82 ± 5.00	67.91 ± 5.18
Config 8	$k_{2}+k_{3}$	0.6960 ± 0.1170	69.47 ± 12.78	66.12 ± 8.68	67.34 ± 9.88
Config 9	$k_{2}+k_{4}$	0.8120 ± 0.0329	**81.05 ± 4.08**	74.78 ± 2.68	77.05 ± 2.89
Config 10	$k_{3}+k_{4}$	0.6430 ± 0.1031	64.47 ± 8.25	57.76 ± 10.48	60.19 ± 9.49
Config 11	$k_{1}+k_{2}+k_{3}$	0.7020 ± 0.1183	68.49 ± 12.79	66.12 ± 10.50	67.14 ± 11.07
Config 12	$k_{1}+k_{2}+k_{4}$	0.8010 ± 0.0399	75.79 ± 7.53	75.23 ± 3.32	75.43 ± 4.35
Config 13	$k_{1}+k_{3}+k_{4}$	0.6960 ± 0.0488	67.11 ± 3.34	66.12 ± 5.63	66.48 ± 4.62
Config 14	$k_{2}+k_{3}+k_{4}$	0.8270 ± 0.0116	80.53 ± 6.35	76.27 ± 2.77	77.81 ± 2.70
Config 15	$k_{1}+k_{2}+k_{3}+k_{4}$	**0.8310 ± 0.0099**	79.48 ± 3.23	**77.17 ± 2.82**	**78.00 ± 2.03**

The bolded text represents the combination with the highest evaluation metric among all kernel function combinations.

From Figure 5 and Table 2, it can be observed that the four-kernel classifier simultaneously achieved the highest average AUC, specificity, and accuracy, with slightly inferior but more stable sensitivity. Therefore, we consider the four-kernel combination based on the Linear Kernel, Polynomial Kernel, Sigmoid Kernel, and Gaussian Kernel as the optimal kernel combination we sought. This classification model has been named MKSVRB (Multi-Kernel Support Vector Machine Model for Three-Year Recurrence Prediction of Budd-Chiari Syndrome) by us.

### Model performance evaluation

After we selected the four-kernel classifier as our optimal model, we compared it with other commonly used machine learning models, including RF, XGBoost, and KNN, using the same ten rounds of validation approach to further validate its performance. The performance was evaluated based on the average and standard deviation of AUC, sensitivity, specificity, and accuracy of the ten results.

**FIGURE 6:**
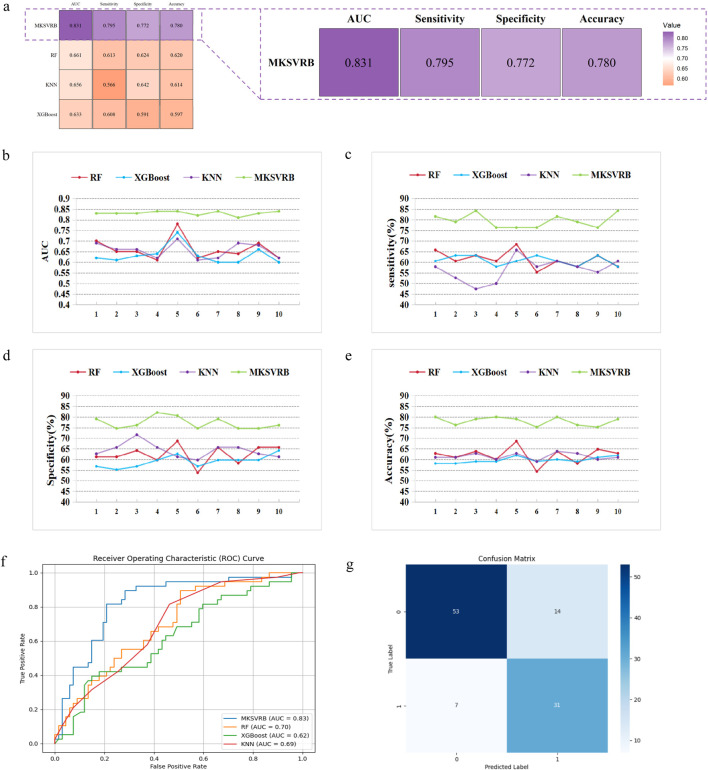
Model performance evaluation. **(a)** Evaluation and comparison of models were conducted using AUC, accuracy, sensitivity, and specificity. **(b–e)** Variations of index of MKSVRB Model and three other machine learning models in ten rounds of validation. **(f)** ROC curves for four machine learning models. **(g)** Confusion Matrix of MKSVRB Model.

Figures 6a–e depicts the trend lines of the evaluation metrics for the four models across the ten validation rounds. From the figure, it is evident that compared to RF, XGBoost, and KNN, our model achieved better results in each metric in every validation round. Table 3 presents the average values and standard deviations of the evaluation metrics for the four models across the ten validation rounds. We can observe that our model exhibits greater advantages over all commonly used machine learning models. Figure 6f displays the ROC curves of the four machine learning models during the first validation round, while Figure 6g shows the confusion matrix of the MKSVRB model during the same validation round, further demonstrating the effectiveness of our approach.

**TABLE 3:** Mean and standard deviation of index of four models.

Models	AUC	Sensitivity (%)	Specificity (%)	Accuracy (%)
RF	0.6610 ± 0.0509	61.32 ± 3.93	62.39 ± 4.44	62.00 ± 3.92
XGBoost	0.6330 ± 0.0424	60.79 ± 2.30	59.11 ± 2.83	59.72 ± 1.42
KNN	0.6560 ± 0.0363	56.58 ± 5.44	64.18 ± 3.45	61.43 ± 1.57
MKSVRB	**0.8310 ± 0.0099**	**79.48 ± 3.23**	**77.17 ± 2.82**	**78.00 ± 2.03**

The bolded text represents the combination with the highest evaluation metric among all models.

To the best of our knowledge, prior to this study, the model constructed by Zhongkai Wang et al. was considered the optimal model in the field of predicting recurrence of Budd-Chiari syndrome. It outperformed traditional scoring models with an AUC of 0.82. Our model achieved an average performance of 0.831 across the ten validation rounds, indicating that our model can be regarded as a more reliable predictor for the recurrence of Budd-Chiari syndrome.

Meanwhile, compared to the AUCs of several prognostic scoring models in previous studies, such as the Child-Pugh score of 0.70, Clichy PI of 0.55, MELD score of 0.67, and Rotterdam score of 0.73, our model also demonstrates advantages (Wang et al., 2023).

### Interpretability of feature importance

**FIGURE 7:**
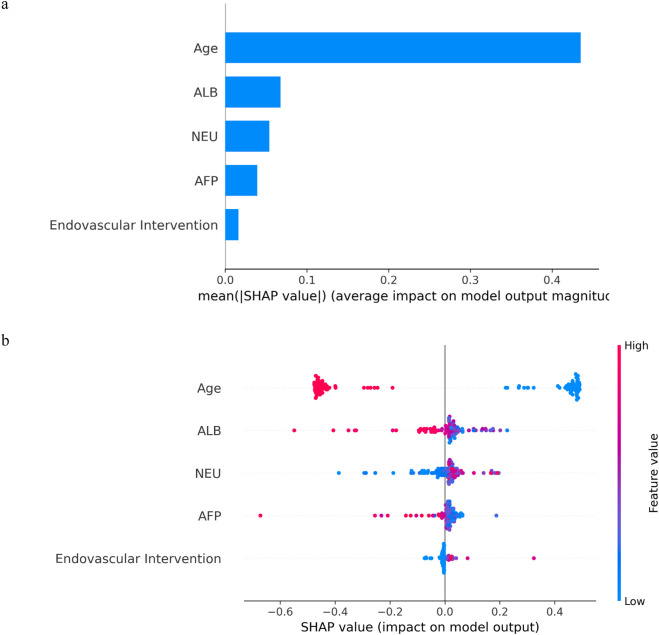
The model’s interpretation: **(a)** The importance ranking of variables according to the mean (|SHAP value|); **(b)** SHAP beeswarm summary plot for impact of each feature in each patient on model output.

Kernel machines, as black-box models, can be effectively interpreted using SHAP values, a method proposed by Lundberg and Lee (2017). In Figure 7a, the mean SHAP values of the MKSVRB model are ranked in descending order to illustrate feature contributions in the test set. The analysis revealed that age exerted the strongest influence on model predictions, followed by ALB, NEU, AFP, and endovascular intervention type. Figure 7b further visualizes the impact of each feature on individual patients in the test cohort. The color gradient of data points reflects feature magnitudes, with red and blue representing values near the maximum and minimum, respectively, and intermediate values in purple. The corresponding SHAP values indicate both the direction and magnitude of each feature’s effect.

As illustrated in the figures, advanced age and elevated ALB levels were identified as protective factors against recurrence in BCS patients, whereas higher NEU levels correlated with an increased likelihood of recurrence. Notably, lower AFP levels also showed a statistical association with recurrence risk. Among endovascular interventions, catheter-directed thrombolysis was associated with a higher recurrence rate compared to simple balloon dilation or stent implantation. The mechanistic underpinnings of these associations are discussed in detail in the second paragraph of the Discussion section.

### Benefits of model application

Decision Curve Analysis (DCA) is a method for evaluating the clinical utility of predictive models in actual clinical decision-making scenarios. In contrast to metrics such as sensitivity, specificity, and the AUC, which measure the diagnostic accuracy of predictive models but fail to account for their clinical utility, DCA offers the advantage of integrating patient or decision-maker preferences into the analysis. It compares the net benefit of different predictive models at specific clinical decision thresholds, including treating all patients or treating none. Net benefit refers to the overall effect considering the benefits and harms of false positives and false negatives (Vickers and Elkin, 2006).

**FIGURE 8:**
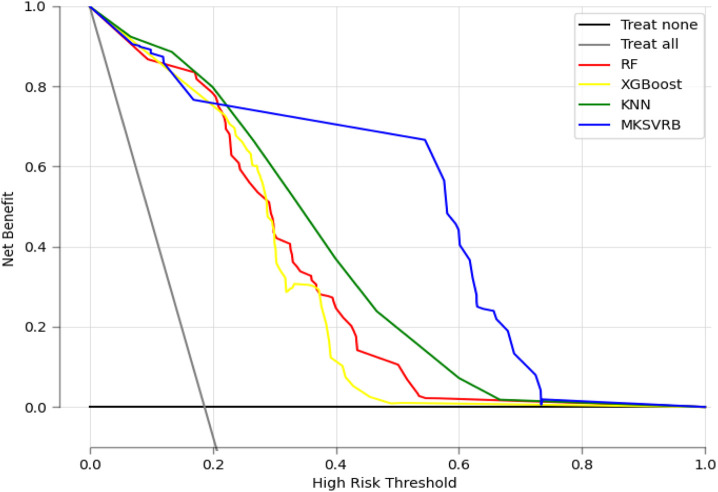
Decision curve analysis of the four prediction models. X-axis indicates the threshold probability for recurrence of BCS and y-axis represents the net benefit gained from intervening on patients with a risk of recurrence at or above the threshold probability. The curve “Treat all” represents the net benefit gained from intervening on all patients while he curve “Treat none” represents the net benefit gained from not intervening on any patients.

In Figure 8, we present the decision curves of our proposed model alongside three other machine learning models. It is evident that our model provides the highest net benefit for decisions regarding recurrence in patients with BCS across the widest range of probability thresholds, demonstrating its significant clinical decision-making utility.

### Deployment of the model

**FIGURE 9:**
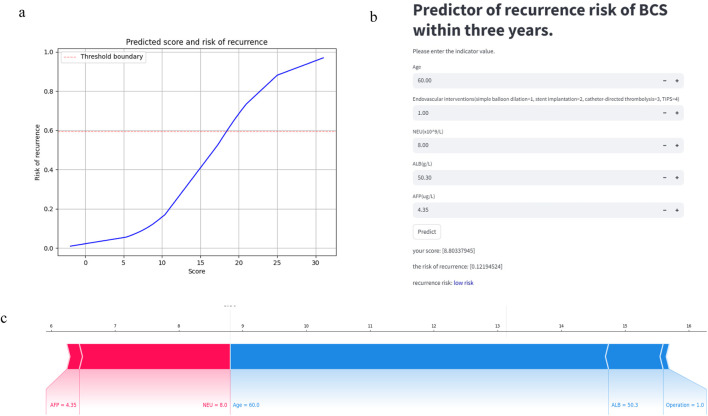
The predictor of recurrence risk of BCS within 3 years: **(a)** The relationship plot between predicted scores and recurrence risk. **(b)** The main part of predictor: Calculate the recurrence risk based on input values. **(c)** The SHAP Force Plot shows the contribution of each patient feature to the risk of recurrence of Budd-Chiari syndrome.

Our model can be accessed at https://mksvrb-5as8kyh4zie.streamlit.app/. It is divided into three sections. Firstly, Figure 9a illustrates the relationship plot between model scores and the risk of recurrence within 3 years, with a risk threshold set at 0.59. Figure 9b functions as a prediction module where patients sequentially input Age, Endovascular interventions, NEU level (x10^9/L), ALB level (g/L), and AFP level (ug/L). By clicking the predict button, the module assesses the risk of recurrence, categorizing it as high risk if above the risk threshold or low risk if below. Due to limitations in the study sample and considerations for accuracy, the predictor currently supports only simple balloon dilation, stent implantation, catheter-directed thrombolysis, and TIPS procedures, excluding others. Figure 9c displays the SHAP Force Plot after risk assessment for each patient, showing how each feature contributes to the risk of Budd-Chiari syndrome recurrence. A red arrow indicates a feature increases recurrence risk, while a blue arrow indicates a decrease. Bar length represents the magnitude of each feature’s effect on recurrence.

## Discussion

In this article, our research introduced the MKSVRB model for predicting the recurrence of Budd-Chiari syndrome with 3 years. Through the detailed experiments encompassing kernel function combinations within our model and comparisons with other machine learning models, we demonstrated its superior performance.

Budd-Chiari syndrome is characterized by its rarity, significant regional variations, and complex etiology, leading to high variability in features (Menon et al., 2004). In terms of model variables, we found that older age may be a protective factor for disease recurrence, which could be related to the fact that Budd-Chiari syndrome is more common in middle-aged individuals (Macnicholas et al., 2012). ALB and NEU are traditional and classical indicators that are widely used in predicting the disease prognosis of liver disease patients. Many studies have confirmed that higher ALB levels are associated with liver function recovery (Su et al., 2019). Liver dysfunction typically causes an increase in AFP levels; however, in extreme cases, such as severe liver failure, the synthesis and release of AFP may decrease. In terms of endovascular interventions, our study found that compared to simple balloon dilation, stent implantation had a lower likelihood of recurrence, while catheter-directed thrombolysis showed a higher risk. The result is similar to a study comparing the long-term prognosis of acute venous thrombosis patients who underwent pharmacomechanical catheter-directed thrombolysis (PCDT) or no PCDT. In this study, there was no significant difference in the recurrence rate between the PCDT group and the no PCDT group within 24 months (Kearon et al., 2019). In another experiment comparing the recurrence rates of catheter-directed thrombolysis and balloon-occluded thrombolysis in patients with Budd-Chiari syndrome, catheter-directed thrombolysis also showed a higher recurrence rate. This higher recurrence rate may be related to the persistent microthrombi on the stent wall, which could serve as a focus for recurrence (Mukund et al., 2024).

Consequently, common machine learning models, including RF, XGBoost, and KNN, struggle to provide accurate predictions for recurrence of Budd-Chiari syndrome. The EasyMKL algorithm,as proposed by Fabio Aiolli and Michele Donini, proves effective for making predictions on small, noisy datasets (Aiolli and Donini, 2015). It aligns well with our data compared to other MKL algorithms. By jointly optimizing internal parameters for kernel functions and the balance parameter in EasyMKL, we combined the PSO algorithm with EasyMKL algorithm, and finally achieved satisfactory results.

On the other hand, we have explored the selection of internal kernel function combinations in SVM for predicting recurrence of Budd-Chiari syndrome.By comparing combinations of four common kernel functions, we found that the Linear Kernel performed best among single-kernel SVMs, but a composite kernel function based on all four base kernels was more suitable for the interrelation of features in Budd-Chiari syndrome data. In terms of combining multiple kernels, our research found that convex combinations of multiple kernels are not always effective. The Linear Kernel performed the best among all base kernels, but the performance of the combination with the Sigmoid Kernel, Config 6, was far worse than either kernel alone, nearly achieving the lowest rating among all kernel combinations. Similarly, Config 8 and Config 10 also performed much worse than their constituent base kernels. This phenomenon was also observed in three-kernel combinations: Config 11 and Config 13 performed lower than any single base kernel in their composition.

However, this does not imply that multi-kernel learning itself is flawed. Config 9, Config 12, Config 14, and ultimately our desired four-kernel classifier are excellent success cases of multi-kernel learning, where combinations of base kernels have created more effective new hybrid kernels. Additionally, Config 5 and Config 7 produced more moderate performance between the two base kernels.The question of why strong kernel combinations can pruduce weak hybrid kernels, as well as potentially stronger kernels, and why the same is true for weak kernel combinations, remains an unsolved problem in machine learning. Internal kernel machines are still considered a black box, making this issue difficult to explain. Kernel tricks cleverly calculate the inner product of data features in an infinite-dimensional feature space, which we typically cannot fully imagine or position (Johnson et al., 2020).

In addition, we have conducted clinical decision analysis to assess the practical clinical utility of the model. After we have determined that the model would contribute to clinical decision-making, we deployed it online for use by all doctors and patients in need. We aim for our model to assist in evaluating Budd-Chiari syndrome patients with high-risk recurrence factors, supporting personalized treatment and prognosis decisions for each patient.

## Conclusion

In this paper, we have explored risk factors influencing relapse of BCS patients and proposed a MKSVRB model that effectively predicts the recurrence of BCS patients within 3 years. Experimental results demonstrate that our model outperforms previous prediction methods and other machine learning models, demonstrating significant potential for clinical application. We hope that our model will contribute to prognosis decision support and recurrence prevention for Budd-Chiari syndrome patients.

## Data Availability

The original contributions presented in the study are included in the article/supplementary material, further inquiries can be directed to the corresponding authors.
